# Volatile Compounds Analysis and Biomarkers Identification of Four Native Apricot (*Prunus armeniaca* L.) Cultivars Grown in Xinjiang Region of China

**DOI:** 10.3390/foods11152297

**Published:** 2022-08-01

**Authors:** Cai Zhao, Jinkui Sun, Xilei Pu, Xuewei Shi, Weidong Cheng, Bin Wang

**Affiliations:** Food College, Shihezi University, Xinjiang Uygur Autonomous Region, Shihezi 832000, China; 20202011018@stu.shzu.edu.cn (C.Z.); sjk767157919@163.com (J.S.); puxilei@stu.shzu.edu.cn (X.P.); shixuewei@shzu.edu.cn (X.S.); cwd0221@163.com (W.C.)

**Keywords:** apricot (*Prunus armeniaca* L.), volatiles, biomarkers, HS-SPME-GC-MS/MS, multivariate analysis

## Abstract

Flavor (odor and taste) have a significant role in the consumer’s acceptance, and volatile compounds are responsible for the odor of apricots. In the present work, headspace solid-phase microextraction with gas chromatography coupled to tandem mass spectrometry (HS-SPME-GC-MS/MS) together with multivariate analysis, i.e., partial least square discrimination analysis (PLS-DA), were applied to construct the volatile fingerprints and biomarkers of apricots in Xinjiang, China. As a result, a total of 63 volatile substances were identified in the fruits of four apricot cultivars, seven of which were considered to serve as volatile biomarkers, which are damascenone for Dabaiyou apricots; acetophenone, myrcenol and 7-hexadecenal for Luopuhongdaike apricots; 2,4-dimethyl-cyclohexanol for You apricots; eucalyptol and salicylaldehyde for Xiaobai apricots. Moreover, Xiaobai apricots were richer in soluble sugars, organic acids and total phenolic and total flavonoid content than the other three apricot varieties. This work helps to characterize the volatile profiles and biomarkers of different apricot cultivars while providing theoretical guidance for developing apricot-flavored foods in practical production.

## 1. Introduction

Apricot (*Prunus armeniaca* L.), a delicious table fruit, originated from China and later spread to Europe [1]. Nowadays, apricots were consumed worldwide, especially in Mediterranean countries such as Italy, France and Spain [1,2]. Due to its pleasant flavor and abundant nutrition, apricots receive wide acceptance by consumers. In addition to being consumed as a fresh fruit, apricots have been processed into juices, pulps, jellies, etc. [3]. Due to its superior climate and characteristics of its geographical position suitable for apricot growth, Xinjiang has become one of the largest apricot-producing areas in China [4]. Apricot output in Xinjiang reached 933,088,000 kg in 2018, and has become an important part of the income for local cultivators [5]. At present, apricots planted in Xinjiang include almost all the cultivars in China, but only a few species, such as Luopuhongdaike apricots, Xiaobai apricots and Semaiti apricots, are indigenous to the Xinjiang region [6].

As a table fruit, apricot possesses a palatable taste and a pleasant aroma. It was believed that the content of soluble sugars and organic acids contributed to fruit taste [7]. The concentrations of sugars and organic acids were greatly affected by apricot cultivar and maturity [1]. In addition, aroma compounds affected by cultivar and maturity also play a crucial role in the overall flavor and commercial value of apricots [8,9]. Headspace solid-phase microextraction combined with gas chromatography–tandem mass spectrometry (HS-SPME-GC-MS/MS) has been widely used to identify volatile compounds in various fruits [10,11].

Besides delicious flavor, apricots contain a variety of nutritive compounds. There are 0.4 g proteins and 0.1 g lipids/100 g of fresh weight [12]. Apricot is also a rich source of soluble sugars, fiber, mineral elements (especially K, Na, Ca, Mg, Fe, P, Zn, Cu and Se) [13], vitamins (vitamin A, vitamin C, pantothenic acid, thiamine, riboflavin and niacin) [14,15] and carotenoids (β-carotene, β-cryptoxanthin and γ-carotene) [16]. In addition, apricots have been shown to exert antioxidant activity, which is beneficial for human health [17]. In particular, polyphenols in apricot are one of the main sources of antioxidant activity. In a previous study, it was demonstrated that rutin, chlorogenic acids, (+)-catechin and (−)-epicatechin were the dominant phenolic compounds in apricots [18].

With the development of extraction and detection techniques, more than 300 volatile compounds have been characterized from various apricot cultivars in the southern Xinjiang region of China [5]. These volatiles were grouped into alcohols, acids, esters, aldehydes, lactones and terpenes based on their functional components. The difference in volatiles among the apricot varieties was very clear. However, there has been less work to find the volatile biomarkers responsible for the flavor in a given apricot cultivar. In the present study, HS-SPME-GC-MS/MS was used to analyze volatile compounds from four native apricot cultivars from Xinjiang. The biomarkers of the characteristic flavor in apricot were subsequently predicted based on multivariate analysis of volatile compounds. The result will provide much more useful information related to the volatile profile of apricots and help guide the practical production of apricot-flavored foods.

## 2. Materials and Methods

### 2.1. Raw Fruit Materials

Four native apricot cultivars (Dabaiyou, DBY; Luopuhongdaike, LPH; You, YOU; Xiaobai, XB) were grown in the Heshuo region, Xinjiang (42°23’N, 86°84’E, 1609 m above sea level; monthly average precipitation: 8.7 mm; monthly average temperature: 26.5 °C, monthly average humidity: 39.5%). Samples were harvested at commercial maturity with a firmness of 9.0–12.0 N by a digital display fruit sclerometer (GY–4; Zhejiang Tup Instrument Co., Ltd., China) in June 2019. Sixty fruits per tree were collected from five different trees planted in the same orchard. Apricots were sampled on the basis of uniformity of size and color. A total of 300 apricot fruits of each cultivar were randomly divided into three replicates. The samples were transported to the laboratory at once. The fresh juice was produced by a juicer (PHILIPS HR1889/71, Shanghai, China) for physicochemical analysis within each replicate.

### 2.2. Determination of Total Soluble Solids and Titratable Acidity

Total soluble solids were analyzed by using a digital refractometer (Atago PR-101R, Tokyo, Japan). The titratable acidity was expressed as percent malic acid per 100 g, whose content was titrated to pH 8.1 with NaOH (0.1N). All analyses were performed in triplicate.

### 2.3. Determination of Total Phenolic and Total Flavonoid Content

Fresh apricots were cleaned with distilled water and the stones were removed. The samples were frozen and lyophilized using a vacuum freeze dryer (Christ Alpha 2-4, Osterode, Germany) for 24 h. Dried apricots were ground using a mortar to obtain apricot powder, then stored at −80 °C until analysis. The polyphenol extracts were prepared using the method previously described by Dulf et al. [19] with small modifications. A 1 g powder sample was extracted with 20 mL of extraction mixture (1:80:19, hydrochloric acid/methanol/water) in a 40 °C ultrasonic bath for 30 min. Then, the mixture was centrifuged at 8000× *g* for 10 min, and the filtrate was centrifuged under the same conditions once again. The resulting extract was evaporated to dryness under a vacuum and then dissolved in methanol. The polyphenol extract was stored at 4 °C until the determination of phenolic and flavonoid content.

Total phenolic content was analyzed based on a previous method, as described earlier by Bakar et al. [20] with some modifications. Briefly, two hundred microliters of polyphenol extract were mixed with distilled water until the volume was 10 mL. Then, 2.25 mL of Folin–Ciocalteu reagent was added to the colorimetric tube. After 5 min at room temperature, 2.25 mL of sodium carbonate (60 g/L) solution was transferred to the mixture. After standing in the dark at room temperature for 90 min, absorbance was measured at 765 nm using a spectrophotometer (T3200; Shanghai, China). Results were expressed as gallic acid equivalents per 100 g of dry mass (mg GAE/100 g).

Total flavonoid content was determined according to the previous technique published by Fan et al. [21] with slight modifications. Briefly, 2.5 mL of phenolic extracts was added to 7.5 mL of ethanol (60%, *w*/*w*) solution and mixed with 0.3 mL of NaNO_2_ (5%, *w*/*w*) solution. After 6 min, 0.6 mL of AlCl_3_ ∙6H_2_O (10%, *w*/*w*) solution was added to the mixture. Then, after standing for 5 min, 1 mL of NaOH (1.0 mol/L) was added. The absorbance was measured at 510 nm and the results were expressed as rutin equivalents per 100 g of dry mass (mg RE/100 g).

### 2.4. Determination of Soluble Sugars and Organic Acids

Standard references for sugars and organic acids were acquired from Sangon Biological Reagent Company (Shanghai, China). The extraction and analysis of sugars were performed based on a previous method [6]. The organic acids were determined using a previous method described by Chen et al. [22] with some modifications. Briefly, the 1 mol/L K_2_HPO_4_ solution and 3% methanol were employed as the mobile phases in the HPLC system (Agilent 1200, Palo Alto, CA, USA). The flow rate was 0.5 mL/min. The sample was separated using a Dikma C18 (4.6 mm × 250 mm, 5 μm, Guangzhou lvbaicao Scientific Instrument Co., Ltd., Guangzhou, China) column and detected using a UV detector at an optimum wavelength of 210 nm. The quantification of sugars and organic acids was performed according to a calibration curve, which was prepared using commercial standards. The results were expressed as grams per 100 g of fresh weight (FW).

### 2.5. Determination of Volatile Compounds

The volatiles were extracted based on the Aubert et al. [1] technique with slight modifications. Briefly, 3.5 g of frozen apricot tissue was combined with 5 mL of calcium chloride in a vial with a lid (20 mL). Then, 0.5 g sodium chloride and 1 μL of 2-octanol solution were placed into the mixture. The mixed solution was equilibrated at 50 °C for 10 min and extracted at the same temperature for 50 min using an SPME fiber coated with divinylbenzene/carboxen/polydimethylsiloxane (DVB/CAR/PDMS, 50/30 μm) (Supelco, Sigma–Aldrich Corp., St. Louis, MO, USA). After extracting, the fiber was inserted into the gas chromatography (GC) injector. The desorbed volatiles were managed in split-less mode at 230 °C for 5 min.

The GC-MS/MS detection for volatile compounds in apricot was performed using an Agilent 8890B GC equipped with an Agilent 7000 MS detector (Agilent Technologies Inc., Palo Alto, CA, USA) and an HP Innowax (30 m × 0.25 mm × 0.25 μm, Agilent, USA) fused-silica capillary column. The injection port temperature was 230 °C and helium was used as the carrier gas at a rate of 1.0 mL/min. The oven temperature was initially set at 40 °C for 2 min, and gradually increased to 180 °C at a rate of 3 °C per min, holding there for 3 min. For MS/MS detection, an ionization source of 270 °C and an electron energy of 70 eV were used. Total ion chromatographs (TICs) were generated in a scanning range from m/z 35 to 350 at a rate of 5 scans/s. Volatile components were identified first using NIST 98 and Wiley 6 mass spectral libraries. The retention index (RI) values were used to further identify each compound. The quantitative analysis of compounds was performed using the internal standard (2-octanol).

### 2.6. Statistical Analysis

Origin (version 8.5, Northampton, MA, USA) was used to create the bar chart, scatter plot and chromatograms. Partial least square discriminant analysis (PLS-DA) is a discriminant analysis method based on multivariable data. The PLS-DA model was used as a regression method for supervised clustering, which can effectively distinguish the observed values between groups and find the influencing variables that lead to the difference between apricot cultivars. The PLS-DA and loading graph were performed with the software SIMCA 14.1. The Venn diagram and heatmap of compounds were analyzed using TB tools. All data were analyzed by one-way analysis of variance (ANOVA) using SPSS 19.0 (SPSS Inc., Chicago, IL, USA). The significant differences were assessed with Duncan’s multiple range tests (*p* < 0.05).

## 3. Results and Discussion

### 3.1. Determination of Total Soluble Solids and Titratable Acidity in Four Different Apricot Cultivars

Soluble solids content and titratable acidity were considered as the most influential factors for fruit quality. The soluble solids content of DBY, LPH, YOU and XB apricots differed significantly (*p* < 0.05), as shown in Table 1. The results were similar to other apricot cultivars described in the previous study. More specifically, the ‘Goldrich’ apricots showed a soluble solids content of 11% and the ‘Iranien’ cultivar showed about 16% [23]. In this work, XB apricots showed the highest soluble solids content (18.58%), followed by LPH apricots, which accounted for 15.63%. The lowest concentration of soluble solids content was found in DBY apricots, which accounted for 6.56%. The titratable acidity of the four apricot varieties ranged from 0.12% to 0.36%, while it was observed that titratable acidity ranged from 0.41% to 0.66% in previous work [5]. In addition, XB apricots only had 0.12% titratable acidity, so XB showed a difference in relation to the other three cultivars.

### 3.2. Total Phenolic and Total Flavonoid Content in Four Different Apricot Cultivars

Flavonoids, which are polyphenol compounds, are ubiquitous in the fruits and vegetables of the human diet [24]. In this work, the content of total phenolic and total flavonoid showed a significant difference (*p* < 0.05) between apricot varieties (Table 1). Total phenolic content ranged from 36.11 to 66.36 mg GAE/100 g and total flavonoid content ranged from 22.67 to 45.57 mg RE/100 g in four apricot varieties from Xinjiang. The total phenolic content and total flavonoid content were lower compared to 15 apricot cultivars planted in the south Moravian region [25]. Moreover, the total phenolic and total flavonoid contents in XB apricots were the highest (66.36 mg GAE/100 g and 45.57 mg RE/100 g, respectively) among investigated varieties. Flavonoids have natural antioxidant and anticancer capacities [26]. Therefore, it is possible that XB apricots have good properties to be used in folk medicine [27].

### 3.3. Composition of Sugars and Organic Acids in Four Different Apricot Cultivars

Sugars and organic acids are considered as important primary metabolites that are correlated to nutrition and odor of apricots. Fructose, sorbitol, glucose and sucrose were reported as the major sugars in the fresh and dried apricot samples. These sugars were also identified in this work. It was obvious that the contents of sucrose in four apricot samples were the highest (Table 2) compared to other sugars, which were 42.49, 63.02, 30.92 and 77.58 mg/g FW in DBY, LPH, YOU and XB cultivars, respectively. Previous finding also proved that sucrose was more important than fructose and glucose in five commercial apricot genotypes from India [28]. Fructose content ranged from 9.52 to 16.58 mg/g FW in the four apricot varieties. XB apricots had the maximum sorbitol content (11.04 mg/g FW), followed by LPH (10.43 mg/g FW), YOU (6.27 mg/g FW) and DBY apricots (4.47 mg/g FW). For glucose, the content in DBY, LPH, YOU and XB apricots was 12.18, 24.82, 13.44 and 28.89 mg/g FW, respectively.

Malic acid was found to be the predominant organic acid in Malatya apricot varieties [29]. This result was also found in this study. Malic acid is considered the main organic acid and showed a significant difference (*p* < 0.05) in the four apricot cultivars (Table 3). The highest concentration of malic acid was determined in XB apricots, followed by YOU, DBY and LPH apricots (1077.98, 616.74, 245.63 and 104.59 mg/100g FW, respectively). For quinic acid, XB apricots showed the highest level (193.21 mg/100g FW) and DBY apricots had the lowest content (49.58 mg/100g FW). The content of ascorbic acid varied from 38.20 to 7.73 mg per 100g FW in XB and LPH apricots, respectively. The citric acid concentration in DBY and XB apricots was higher than in YOU and LPH apricots (241.26, 233.75, 186.45 and 91.82 mg/100g FW, respectively). Previous work also reported a similar result [30]. The content of succinic acid in YOU, XB, LPH and DBY apricots accounted for 9.74, 8.27, 6.35 and 5.37 mg/100g FW, respectively. The content of sugars and organic acids also varied with the maturity of apricot. Sugar content increased while organic acid content decreased from commercial ripe to tree ripe stages [31].

### 3.4. Volatile Profiles of Fruits in Four Different Apricot Cultivars

**Figure 1:**
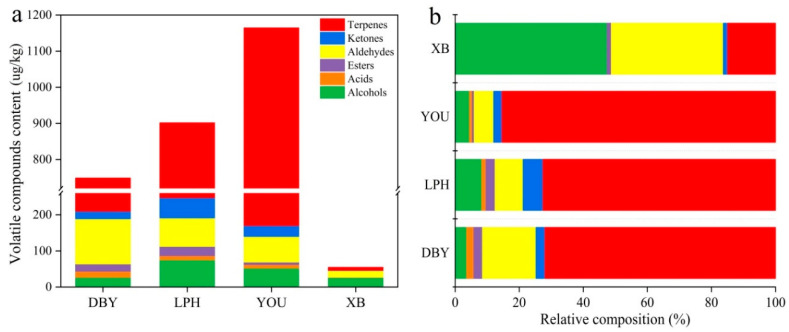
The content (**a**) and relative composition (**b**) of volatile compounds in four different apricot cultivars. DBY, Dabaiyou apricot; LPH, Luopuhongdaike apricot; YOU, You apricot; XB, Xiaobai apricot.

**Figure 2:**
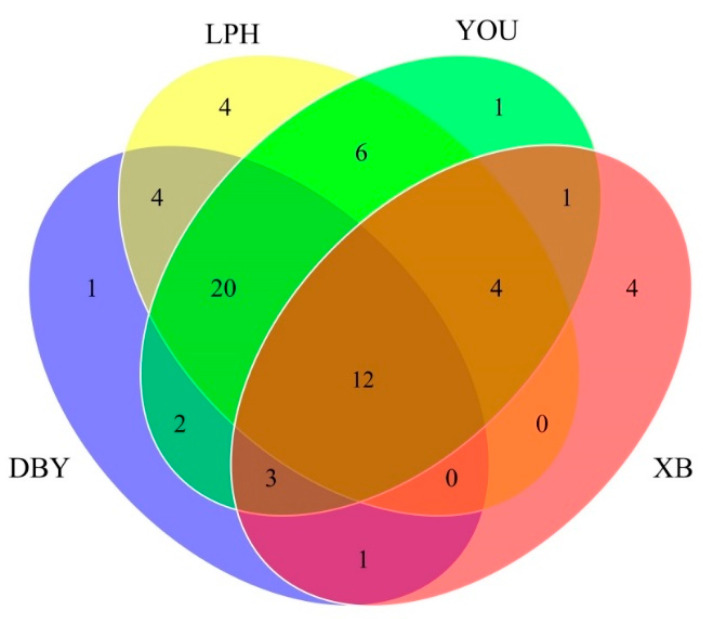
The Venn diagram of volatile compounds in four different apricot cultivars. DBY, Dabaiyou apricot; LPH, Luopuhongdaike apricot; YOU, You apricot; XB, Xiaobai apricot.

It is known that volatile compounds play a dominant role in contributing to the overall flavor of fruits. HS-SPME-GC-MS/MS was used to detect volatile compounds in four different apricot cultivars (Figure S1). A total of 63 volatile compounds were identified from the four apricots. According to their functions, compounds were grouped into 14 alcohols, 6 acids, 6 esters, 15 aldehydes, 13 ketones and 9 terpenes (Table S1). These volatile classes were considered to be the central components that distinguished plums from apricots [32,33]. The result was different from a previous study that found 208 volatile substances in 14 apricot cultivars from the Xinjiang region [5]. In this study, the total content of volatiles in the YOU apricots was the highest, followed by the LPH, DBY and XB apricot varieties (Figure 1a). Moreover, it was observed that the composition of volatile compounds was different among the four apricot varieties (Figure 1b). The proportion of terpenes in DBY, YOU and LPH apricots was relatively higher among overall compounds. It was noteworthy that alcohols and aldehydes were the dominant volatile compounds in XB apricots, which led to considerable differences between XB apricots and the other three apricot samples. The results suggested that the composition and content of volatiles clearly varied in different apricot varieties. However, there were still 12 volatiles that occurred in all four apricot cultivars (Figure 2).

In addition, the alcohol compounds with a high concentration were ethanol and 1-dodecanol, which were present in all four apricots (Table S1). The concentrations of ethanol and 1-dodecanol were more than 2.00 μg/kg in all apricot cultivars except 1-dodecanol in XB apricots. It is worth mentioning that 2-decen-1-ol, 1-octanol, myrcenol, nonanol and 2,4-dimethyl-cyclohexanol were only found in the LPH, XB, LPH, XB and YOU cultivars, respectively. Interestingly, no acids in the XB apricots were detected, but they were generally present in the other three apricots. The ester compound with the highest level was ethyl acetate, with an excess of 21.00 μg/kg in LPH apricots. It has been reported that the maximum content of hexanal in some apricot cultivars was 1475.30 μg/kg [34]. In this work, the highest concentration of hexanal was only 2.37 μg/kg in DBY apricots. Another C6 aldehyde (i.e., 2-hexenal) was detected at a higher level. The content of 2-hexenal exceeded 12.00 μg/kg in the four apricots. Those C6 compounds were considered important contributors to the green and fresh notes [34]. 2-Pentenal was abundant in the DBY, LPH and YOU cultivars (40.31, 9.81 and 6.50 μg/kg, respectively) and was considered the major aldehyde compound in this study. It is possible that some volatile compounds at low levels may also have an important impact on the flavor of apricot [35]. A lower amount of benzaldehyde, involved in basic floral and fruity notes [36], was found in the four apricot cultivars. It was reported that benzaldehyde was an important component of essential oil in Japanese apricots [37]. The most abundant ketone recorded by previous work was 3-hydroxy-2-butanone [38], which was not in agreement with our research. In this work, 3-hydroxy-2-butanone was not detected in the four apricot cultivars. The contents of linalool and α-terpineol all reached up to 190.00 μg/kg and above in the DBY, LPH and YOU cultivars, while these compounds were present in XB apricots with a low concentration. Previous work suggested that linalool contributes to citrus, lemon, fragrant and sweet flavors, while α-terpineol provides fragrant, floral as well as lilac notes. β-linalool was the main terpene compound in many apricot cultivars from Romania [39]. Aubert et al. [1] also described that linalool and α-terpineol were responsible for the flowery note in apricots.

### 3.5. Partial Least Square Discrimination Analysis of Volatile Compounds

**Figure 3:**
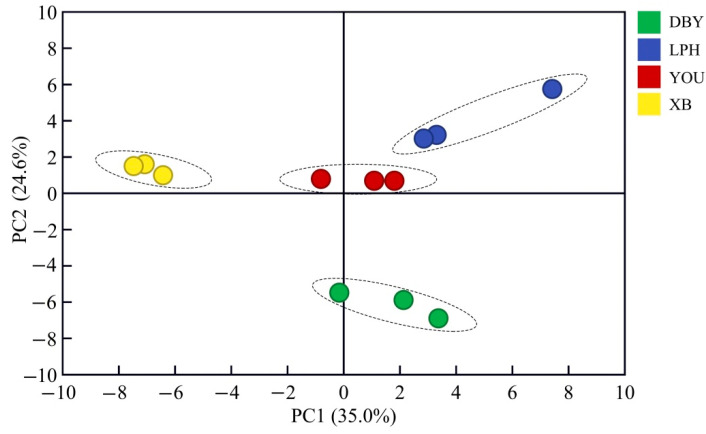
Partial least square discrimination analysis (PLS-DA) of volatile compounds in four different apricot cultivars. DBY, Dabaiyou apricot; LPH, Luopuhongdaike apricot; YOU, You apricot; XB, Xiaobai apricot.

Multivariate analysis has been used successfully to distinguish varieties of apricot [40]. In the present study, partial least square discrimination analysis (PLS-DA) was applied to graphically classify sample groups based on similarity and/or dissimilarity of volatile compounds in the four apricot cultivars [41]. As indicated in Figure 3, the total cumulative variance of 59.6% was contributed to by the two principal components (PC1 and PC2) from volatile compounds, and they accounted for 35.0% and 24.6% of the total variance, respectively. Furthermore, it was obvious that the four apricot varieties were well separated. The result suggested that there were clear differences among the four different apricot cultivars. The loading graph (Figure S2) provided the distribution of volatile compounds of four apricots. XB apricot was characterized by 1-octanol, nonanol, salicylaldehyde and eucalyptol. There was a positive correlation between XB apricots and these four volatiles. Furthermore, the DBY apricot was separated from the other three apricot varieties along PC2 (Figure 3). The characteristics of the PLS-DA model strongly corresponded to the above results for compounds. In conclusion, the obvious difference in volatile compounds among the different apricot varieties provided a great basis for identifying the biomarkers for the four apricot cultivars.

### 3.6. Identification of Volatile Biomarkers for Four Different Apricot Cultivars

**Figure 4:**
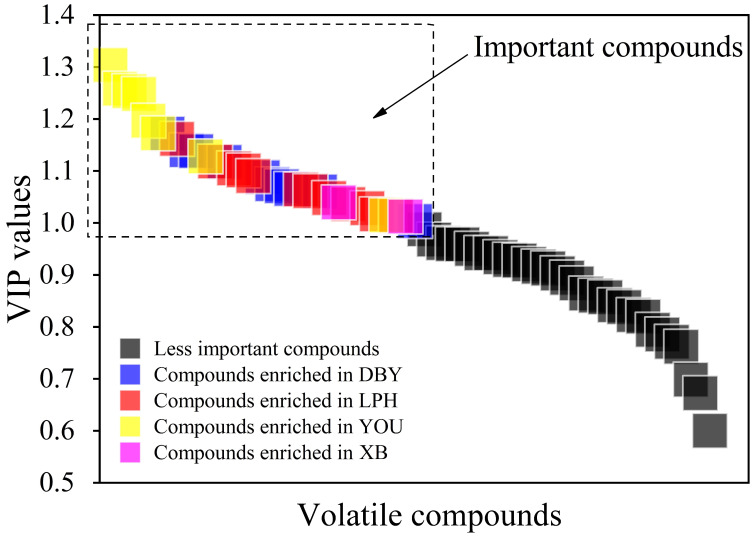
The variable importance for the projection in PLS-DA model. DBY, Dabaiyou apricot; LPH, Luopuhongdaike apricot; YOU, You apricot; XB, Xiaobai apricot.

**Figure 5:**
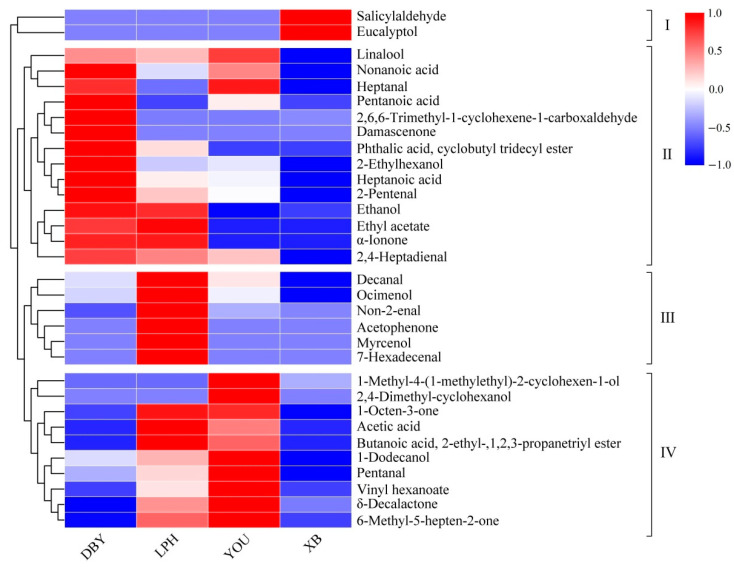
The heatmap analysis of the volatile compounds (VIP > 1) in four different apricot cultivars. DBY, Dabaiyou apricot; LPH, Luopuhongdaike apricot; YOU, You apricot; XB, Xiaobai apricot.

The variable importance for the projection (VIP) was found to distinguish the most characteristic variables based on the result of the PLS-DA classification above, and the VIP plot is given in Figure 4. Generally speaking, a VIP value greater than 1.0 means significance to the overall volatile profile, whereas a VIP value less than 0.5 shows its unimportance [42]. In this study, the VIP values larger than 1.0 were picked out to produce the volatile clustering heatmap of the four apricot cultivars (Figure 5 and Figure S3). According to the row clustering results, the compounds (VIP > 1.0) were divided into four categories. It was obvious that the relative contents of salicylaldehyde and eucalyptol in XB apricots were the most abundant in the first group. Category II included 14 volatiles. Most of these compounds in the DBY apricots were richer than in the other three cultivars. In particular, the third category included decanal, ocimenol, non-2-enal, acetophenone, myrcenol and 7-hexadecenal, and their relative content was higher in LPH apricots compared to the other apricot cultivars. In the 4th category, most compounds were relatively abundant in LPH and YOU cultivars except for 1-methyl-4-(1-methylethyl)-2-cyclohexen-1-ol and 2,4-dimethyl-cyclo-hexanol.

The seven differentiated volatile compounds whose VIP > 1.0 are shown in Table 4. Particularly, damascenone only existed in DBY apricots. 2,4-Dimethyl-cyclohexanol was only present in YOU apricot samples. Three compounds, i.e., acetophenone, myrcenol and 7-hexadecenal, were only found in LPH apricots. Moreover, eucalyptol and salicylaldehyde were characteristic volatiles for XB apricots.

## 4. Conclusions

The volatile fingerprints of four native apricot cultivars planted in the Heshuo area of Xinjiang, China have been identified using HS-SPME-GC-MS/MS. Moreover, the multivariate analysis, i.e., partial least square discrimination analysis (PLS-DA) model, was successively performed to discriminate the differences in volatile compounds and provided a good cluster to classify the volatile substances of the different apricot cultivars. The biomarkers of volatiles in each apricot were found based on the variable importance for the projection analysis. As a result, a total of 63 volatile compounds, including 14 alcohols, 6 acids, 6 esters, 15 aldehydes, 13 ketones and 9 terpenes, were acquired. Seven characteristic components could be taken as biomarkers of volatile compounds in four apricot cultivars, which were damascenone for Daibaiyou apricots; acetophenone, myrcenol and 7-hexadecenal for Luopuhongdaike apricots; 2,4-dimethyl-cyclohexanol for You apricots; eucalyptol and salicylaldehyde for Xiaobai apricots. Furthermore, the highest concentrations of sucrose and malic acid were present in Xiaobai apricots. Soluble solids content, total phenolic content and total flavonoid content of Xiaobai apricots were higher than those determined in Dabaiyou, You and Luopuhongdaike apricot cultivars. This study not only provides a feasible approach to analyzing the characteristic volatile compounds from different apricot cultivars but also sets up a theoretical basis for developing apricot-flavored foods in practical production.

## Figures and Tables

**Table 1:** Fundamental physicochemical properties in four different apricot cultivars.

Apricot Cultivar	Total Soluble Solids (%)	Titratable Acidity (%)	Total Phenolic Content (mg GAE/100 g)	Total Flavonoid Content (mg RE/100 g)
DBY	6.56 ± 0.13 ^d^	0.34 ± 0.02 ^a^	43.68 ± 0.89 ^c^	28.69 ± 2.69 ^c^
LPH	15.63 ± 0.06 ^b^	0.16 ± 0.02 ^b^	56.57 ± 2.55 ^b^	34.85 ± 2.54 ^b^
YOU	8.60 ± 0.11 ^c^	0.36 ± 0.01 ^a^	36.11 ± 2.06 ^d^	22.67 ± 3.14 ^d^
XB	18.58 ± 0.16 ^a^	0.12 ± 0.01 ^c^	66.36 ± 0.23 ^a^	45.57 ± 0.63 ^a^

The different superscripts in the column mean significant differences (*p* < 0.05, Duncan’s test) for different apricot cultivars. Values are presented as mean ± standard deviation (*n* = 3). DBY, Dabaiyou apricot; LPH, Luopuhongdaike apricot; YOU, You apricot; XB, Xiaobai apricot.

**Table 2:** The composition and content of soluble sugars in four different apricot cultivars.

Apricot Cultivar	Soluble Sugars (mg/g FW)			
	Fructose	Sorbitol	Glucose	Sucrose
DBY	9.85 ± 0.65 ^b^	4.47 ± 0.15 ^c^	12.18 ± 1.67 ^c^	42.49 ± 1.51 ^c^
LPH	15.84 ± 2.10 ^a^	10.43 ± 0.23 ^a^	24.82 ± 2.58 ^b^	63.02 ± 3.52 ^b^
YOU	9.52 ± 0.96 ^b^	6.27 ± 0.69 ^b^	13.44 ± 0.50 ^c^	30.92 ± 2.02 ^d^
XB	16.58 ± 0.93 ^a^	11.04 ± 0.72 ^a^	28.89 ± 2.85 ^a^	77.58 ± 5.36 ^a^

The different superscripts in the column mean significant differences (*p* < 0.05, Duncan’s test) for different apricot cultivars. Values are presented as mean ± standard deviation (*n* = 3). DBY, Dabaiyou apricot; LPH, Luopuhongdaike apricot; YOU, You apricot; XB, Xiaobai apricot; FW, fresh weight.

**Table 3:** The composition and content of organic acids in four different apricot cultivars.

Apricot Cultivar	Organic Acids (mg/100 g FW)				
	Quinic Acid	Malic Acid	Ascorbic Acid	Citric Acid	Succinic Acid
DBY	49.58 ± 2.30 ^d^	245.63 ± 12.85 ^c^	25.18 ± 1.56 ^b^	241.26 ± 24.85 ^a^	5.37 ± 0.33 ^d^
LPH	65.53 ± 3.02 ^c^	104.59 ± 7.52 ^d^	7.73 ± 0.38 ^c^	91.82 ± 2.94 ^c^	6.35 ± 0.20 ^c^
YOU	97.89 ± 2.03 ^b^	616.74 ± 23.18 ^b^	27.17 ± 0.97 ^b^	186.45 ± 30.58 ^b^	9.74 ± 0.56 ^a^
XB	193.21 ± 6.54 ^a^	1077.98 ± 37.84 ^a^	38.20 ± 2.58 ^a^	233.75 ± 13.30 ^a^	8.27 ± 0.24 ^b^

The different superscripts in the column mean significant differences (*p* < 0.05, Duncan’s test) for different apricot cultivars. Values are presented as mean ± standard deviation (*n* = 3). DBY, Dabaiyou apricot; LPH, Luopuhongdaike apricot; YOU, You apricot; XB, Xiaobai apricot; FW, fresh weight.

**Table 4:** The biomarkers for the four different apricot cultivars.

No	Volatile Compounds	RI	Apricot Cultivars			
			DBY	LPH	YOU	XB
1	Acetophenone	1115	−	+	−	−
2	Eucalyptol	1237	−	−	−	+
3	Myrcenol	1585	−	+	−	−
4	2,4-Dimethyl-cyclohexanol	1610	−	−	+	−
5	Salicylaldehyde	1672	−	−	−	+
6	Damascenone	1971	+	−	−	−
7	7-Hexadecenal	2144	−	+	−	−

DBY, Dabaiyou apricot; LPH, Luopuhongdaike apricot; YOU, You apricot; XB, Xiaobai apricot. The ‘+’ means that the volatile compound was detected. The ‘−’ means that the volatile compound was not detected.

## Data Availability

Data is contained within the article or supplementary material.

## Supplementary Materials

The following supporting information can be downloaded at: https://www.mdpi.com/article/10.3390/foods11152297/s1. Figure S1: The chromatograms in the HS-SPME-GC-MS/MS analysis of the four different apricot cultivars. Figure S2: The loading graph of PLS-DA analysis of volatile compounds in four different apricot cultivars. Figure S3: The volatile compounds of VIP > 1 in four different apricot cultivars. Table S1: Concentration of volatile compounds in four apricot cultivars (μg/kg).
