# Supplementary material for: Volatile Compounds Analysis and Biomarkers Identification of Four Native Apricot (Prunus armeniaca L.) Cultivars Grown in Xinjiang Region of China
Source: Foods. 2022 Aug 1;11(15):2297. doi: 10.3390/foods11152297 (PMC9368480; doi:10.3390/foods11152297)
Supplement: Supplementary file 1 [file foods-11-02297-s001.zip › foods-1812794-supplementary.pdf]

Supplementary Information for

Volatile compounds analysis and biomarkers identification of four native apricot (*Prunus armeniaca* L.) cultivars grown in Xinjiang region of China

Cai Zhao, Jinkui Sun, Xilei Pu, Xuewei Shi, Weidong Cheng, Bin Wang*

Food college, Shihezi University, Shihezi 832000, Xinjiang Uygur Autonomous Region, P. R. China.

* Corresponding authors

E-mail addresses:

B. W. : binwang0228@shzu.edu.cn.

Tel.: 86-0993-2058093

Supplementary Figures 3

Figure S1. 3

Figure S2. 4

Figure S3. 5

Supplementary Table 6

Table S1. 7

Supplementary Figures

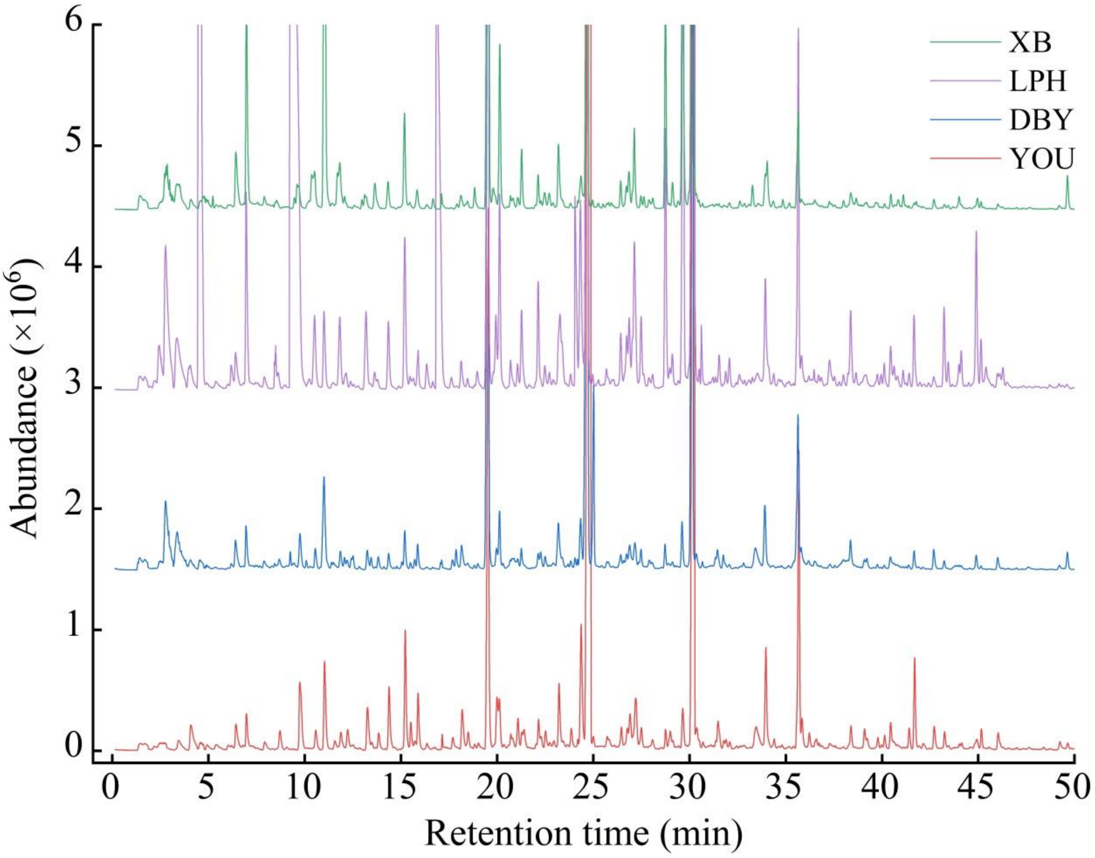

Figure S1.

The chromatograms in the HS-SPME-GC-MS/MS analysis of the four apricot cultivars.

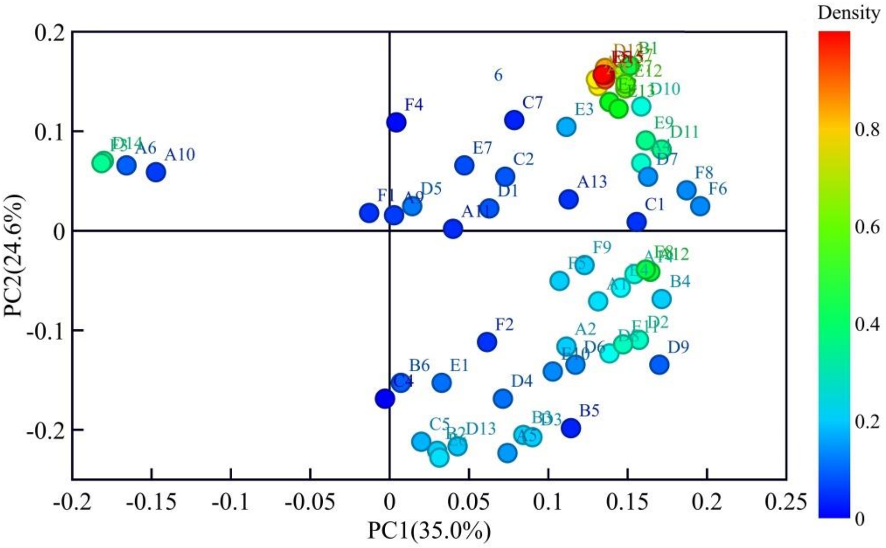

A1: Ethanol
A2: 1-Hexanol
A3: 2-Decen-1-ol
A4: 1-Octen-3-ol
A5: 2-Ethylhexanol
A6: 1-Octanol
A7: 2,8-p-Menthadien-1-ol
A8: Myrcenol
A9: 1-Methyl-4-(1-methylethyl)-2-cyclohexen-1-ol
A10: Nonanol
A11: 2,4-Dimethyl-cyclohexanol
A12: Nerol
A13: 1-Dodecanol
A14: 2,2,6-β-7-Tetramethylbicyclo(4.3.0)nona-1(9),7-dien-5-ol
B1: Acetic acid
B2: Pentanoic acid
B3: Heptanoic acid
B4: Octanoic acid
B5: Nonanoic acid
B6: Decanoic acid

C1: Ethyl acetate
C2: vinyl hexanoate
C3: Butanoic acid, 2-ethyl-, 1,2,3-propanetriyl ester
C4: 2-Ethylhexyl salicylate
C5: Phthalic acid, cyclobutyl tridecyl ester
C6: δ-Decalactone
D1: Pentanal
D2: Hexanal
D3: 2-Pentenal
D4: Heptanal
D5: 2-Hexenal
D6: Octanal
D7: 2-Heptenal
D8: Nonanal
D9: 2,4-Heptadienal
D10: Decanal
D11: Benzaldehyde
D12: Non-2-enal

D13: 2,6,6-Trimethyl-1-cyclohexene-1-carboxaldehyde
D14: Salicylaldehyde
D15: 7-Hexadecenal
E1: 1-Penten-3-one
E2: 1-Octen-3-one
E3: 6-Methyl-5-hepten-2-one
E4: α-Ionone
E5: Acetophenone
E6: Damascenone
E7: Dihydro-β-ionone
E8: 6,10-Dimethyl-5,9-undecadien-2-one
E9: 4-(2,6,6-Trimethyl-1-cyclohexen-1-yl)-3-buten-2-one
E10: β-Ionone
E11: Damascone
E12: 4-(2,6,6-Trimethylcyclohexa-1,3-dienyl)but-3-en-2-one

E13: 5-Hexylfuran-2(3H)-one
F1: β-Myrcene
F2: D-Limonene
F3: Eucalyptol
F4: β-Ocimene
F5: Linalool
F6: 4-Terpineol
F7: Ocimenol
F8: α-Terpineol
F9: Geraniol

Figure S2.

The loading graph of PLS-DA analysis of volatile compounds in four apricot cultivars.

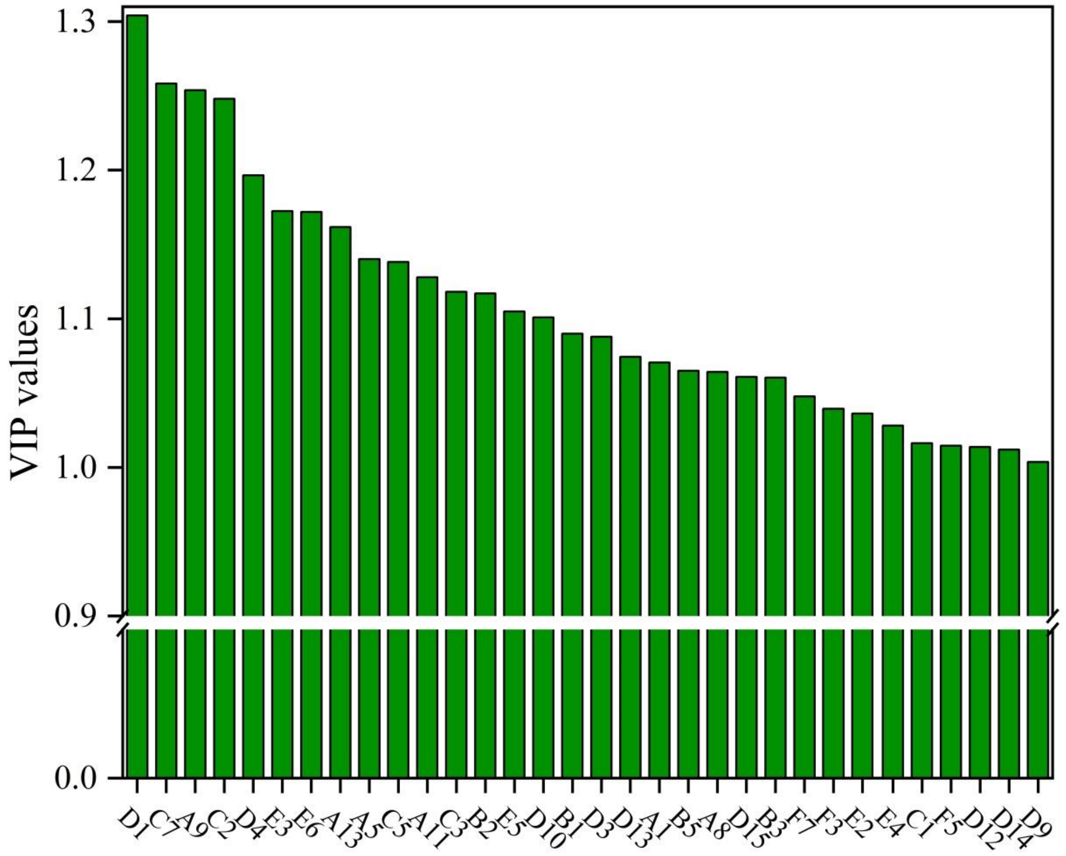

D1: Pentanal
C7: δ-Decalactone
A9: 1-Methyl-4-(1-methylethyl)-2-cyclohexen-1-ol
C2: Vinyl hexanoate
D4: Heptanal
E3: 6-Methyl-5-hepten-2-one
E6: Damascenone
A13: 1-Dodecanol
A5: 2-Ethylhexanol
C5: Phthalic acid, cyclobutyl tridecyl ester
A11: 2,4-Dimethyl-cyclohexanol
C3: Butanoic acid, 2-ethyl-,1,2,3-propanetriyl ester
B2: Pentanoic acid
E5: Acetophenone
D10: Decanal
B1: Acetic acid
D3: 2-Pentenal
D13: 2,6,6-Trimethyl-1-cyclohexene-1-carboxaldehyde
A1: Ethanol
B5: Nonanoic acid
A8: Myrcenol
D15: 7-Hexadecenal
B3: Heptanoic acid
F7: Ocimenol
F3: Eucalyptol
E2: 1-Octen-3-one
E4: α-Ionone
C1: Ethyl acetate
F5: Linalool
D12: Non-2-enal
D14: Salicylaldehyde
D9: 2,4-Heptadienal

Figure S3.

The volatile compounds of VIP>1 in four apricot cultivars.

Supplementary Table

Table S1. Concentration of volatile compounds in four apricot cultivars (μg/kg).

Volatiles categories	Compounds	Apricot cultivars			
		DBY	LPH	YOU	XB
Alcohols	Ethanol	13.31 ± 1.90 [a]	12.30 ± 4.67 [a]	2.23 ± 0.60 [b]	2.86 ± 1.59[b]
	1-Hexanol	1.64 ± 1.42 [a]	0.64 ± 0.57 [a]	1.97 ± 0.88 [a]	nd
	2-Decen-1-ol	nd	20.48 ± 16.07	nd	nd
	1-Octen-3-ol	3.67 ± 3.20	9.31 ± 4.93	nd	nd
	2-Ethylhexanol	3.59 ± 0.35[a]	0.70 ± 0.13[b]	0.82 ± 0.18 [b]	nd
	1-Octanol	nd	nd	nd	22.51 ± 12.94
	2,8-p-Menthadien-1-ol	nd	17.54 ± 9.61	8.58 ± 1.13	nd
	Myrcenol	nd	6.91 ± 2.92	nd	nd
	1-Methyl-4-(1-methylethyl)-2-cyclohexen-1-ol	nd	nd	2.53 ± 1.19	0.17 ± 0.18
	Nonanol	nd	nd	nd	0.11 ± 0.10
	2,4-Dimethyl-cyclohexanol	nd	nd	20.42 ± 13.81	nd
	Nerol	0.82 ± 0.71 [a]	0.98 ± 0.08 [a]	1.04 ± 0.45 [a]	nd
	1-Dodecanol	2.95 ± 0.81 [b]	5.23 ± 4.86 [b]	13.65 ± 3.11 [a]	0.22 ± 0.31 [b]
	2,2,6-β-7-Tetramethylbicyclo(4.3.0)nona-1(9),7-dien-5-ol	1.22 ± 1.07 [a]	1.21 ± 1.05 [a]	1.33 ± 0.51 [a]	nd

Supplementary Table S1. (continued)

Acids	Acetic acid	nd	3.28 ± 0.47	1.66 ± 0.11	nd
	Pentanoic acid	2.70 ± 0.82	nd	0.64 ± 0.33	nd
	Heptanoic acid	2.86 ± 0.47 [a]	1.06 ± 0.97 [b]	0.93 ± 0.20 [b]	nd
	Octanoic acid	5.76 ± 0.44 [a]	5.76 ± 2.68 [a]	3.37 ± 1.02 [a]	nd
	Nonanoic acid	4.07 ± 0.66 [a]	1.30 ± 0.29 [b]	2.54 ± 1.08 [b]	nd
	Decanoic acid	1.05 ± 1.06	nd	0.45 ± 0.40	nd
Esters	Ethyl acetate	15.07 ± 1.64	21.19 ± 12.96	nd	nd
	Vinyl hexanoate	nd	0.68 ± 0.63	2.58 ± 0.41	nd
	Butanoic acid, 2-ethyl-,1,2,3-propanetriyl ester	nd	0.82 ± 0.15	0.57 ± 0.09	nd
	2-Ethylhexyl salicylate	2.69 ± 2.08 [a]	nd	0.30 ± 0.53 [a]	0.20 ± 0.26 [a]
	Phthalic acid, cyclobutyl tridecyl ester	1.05 ± 0.33	nd	nd	nd
	δ-Decalactone	nd	1.41 ± 0.16 [b]	2.71 ± 0.16 [a]	0.39 ± 0.02 [c]
Aldehydes	Pentanal	1.79 ± 0.34 [b]	4.18 ± 1.50 [b]	20.49 ± 2.42 [a]	nd
	Hexanal	2.37 ± 0.90 [a]	1.55 ± 1.48 [a]	1.31 ± 0.16 [a]	nd
	2-Pentenal	40.31 ± 13.09 [a]	9.81 ± 4.07 [b]	6.50 ± 1.26 [b]	nd

Supplementary Table S1. (continued)

	Heptanal	6.92 ± 0.58 [a]	0.83 ± 0.73 [b]	7.64 ± 1.74 [a]	nd
	2-Hexenal	14.89 ± 12.07 [a]	17.25 ± 6.50 [a]	12.99 ± 4.81 [a]	18.43 ± 6.33 [a]
	Octanal	6.68 ± 6.72 [a]	3.22 ± 0.49 [a]	5.44 ± 1.19 [a]	nd
	2-Heptenal	4.98 ± 2.41 [ab]	9.57 ± 4.00 [a]	2.78 ± 1.00 [b]	nd
	Nonanal	3.25 ± 2.82 [a]	1.98 ± 0.61 [ab]	1.85 ± 0.34 [ab]	0.09 ± 0.10 [b]
	2,4-Heptadienal	15.34 ± 3.08 [a]	11.11 ± 1.96 [b]	7.95 ± 1.18 [b]	0.18 ± 0.02 [c]
	Decanal	1.98 ± 0.86 [b]	11.18 ± 0.34 [a]	2.80 ± 0.59 [b]	nd
	Benzaldehyde	1.73 ± 0.69 [b]	4.49 ± 2.72 [a]	1.03 ± 0.54 [b]	0.08 ± 0.07 [b]
	Non-2-enal	nd	2.54 ± 1.37 [a]	0.23 ± 0.22 [b]	0.12 ± 0.12 [b]
	2,6,6-Trimethyl-1-cyclohexene-1-carboxaldehyde	25.10 ± 12.33	nd	nd	0.09 ± 0.11
	Salicylaldehyde	nd	nd	nd	0.09 ± 0.03
	7-Hexadecenal	nd	1.28 ± 0.56	nd	nd
Ketones	1-Penten-3-one	0.69 ± 0.60 [a]	nd	0.79 ± 0.16 [a]	0.22 ± 0.39 [a]
	1-Octen-3-one	0.36 ± 0.42 [b]	8.51 ± 3.08 [a]	7.73 ± 2.91 [a]	nd
	6-Methyl-5-hepten-2-one	nd	3.74 ± 1.57 [b]	6.61 ± 1.51 [a]	0.23 ± 0.07 [c]
	α-Ionone	4.11 ± 2.17	4.29 ± 1.41	nd	nd

Supplementary Table S1. (continued)

	Acetophenone	nd	1.21 ± 0.35	nd	nd
	Damascenone	1.18 ± 0.12	nd	nd	nd
	Dihydro-β-ionone	nd	7.52 ± 11.57	1.23 ± 0.26	nd
	6,10-Dimethyl-5,9-undecadien-2-one	2.96 ± 1.24 [a]	3.25 ± 0.36 [a]	4.56 ± 2.65 [a]	0.07 ± 0.02 [b]
	4-(2,6,6-Trimethyl-1-cyclohexen-1-yl)-3-buten-2-one	0.79 ± 0.70 [a]	2.5 ± 1.51 [a]	0.57 ± 0.60 [a]	nd
	β-Ionone	4.78 ± 2.08 [a]	2.95 ± 3.44 [a]	2.34 ± 0.64 [a]	nd
	Damascone	3.50 ± 1.62 [a]	2.96 ± 1.49 [a]	2.10 ± 0.57 [ab]	0.22 ± 0.23 [b]
	4-(2,6,6-Trimethylcyclohexa-1,3-dienyl)but-3-en-2-one	nd	3.81 ± 2.60	3.21 ± 1.20	nd
	5-Hexylfuran-2(3H)-one	2.54 ± 0.27 [b]	15.33 ± 9.90 [a]	1.12 ± 1.10 [b]	nd
Terpenes	β-Myrcene	2.49 ± 0.62 [a]	2.47 ± 0.97 [a]	4.50 ± 2.63 a	4.03 ± 4.04 [a]
	D-Limonene	5.06 ± 5.26 [a]	nd	8.01 ± 7.23 a	0.33 ± 0.48 [a]
	Eucalyptol	nd	nd	nd	0.26 ± 0.05
	β-Ocimene	nd	0.74 ± 0.04 [a]	1.41 ± 0.84 [a]	0.80 ± 0.94 [a]
	Linalool	291.25 ± 13.88 [ab]	195.74 ± 94.71 [b]	600.22 ± 390.09 [a]	2.39 ± 2.10 [b]
	4-Terpineol	2.89 ± 1.04 [a]	4.72 ± 1.77 [a]	3.43 ± 2.20 a	nd
	Ocimenol	4.08 ± 1.58 [b]	57.14 ± 22.68 [a]	5.11 ± 3.55 b	0.12 ± 0.05 [b]

Supplementary Table S1. (continued)

α-Terpineol	198.42 ± 32.41 [ab]	364.88 ± 157.96 [a]	305.33 ± 212.07 [a]	0.22 ± 0.38 [b]
Geraniol	34.55 ± 1.66 [a]	28.69 ± 26.74 [a]	66.08 ± 46.49 [a]	nd

Notes: Values were presented as mean ± standard deviation (n=3). nd= not detected.

The different superscripts in the column meant significant differences ($P < 0.05$, Duncan's test) for different apricot cultivars.
